# Advancing tools to promote health equity across European Union regions: the EURO-HEALTHY project

**DOI:** 10.1186/s12961-020-0526-y

**Published:** 2020-02-13

**Authors:** Paula Santana, Ângela Freitas, Iwa Stefanik, Cláudia Costa, Mónica Oliveira, Teresa C. Rodrigues, Ana Vieira, Pedro Lopes Ferreira, Carme Borrell, Sani Dimitroulopoulou, Stéphane Rican, Christina Mitsakou, Marc Marí-Dell’Olmo, Jürgen Schweikart, Diana Corman, Carlos A. Bana e Costa, on behalf of the EURO-HEALTHY investigators, Carlota Quintal, João Malva, Lúcio Cunha, Paulo Nossa, Ricardo Almendra, Rui Gama Fernandes, Laia Palència, Lluís Camprubí, Maica Rodríguez-Sans, Mercè Gotsens, Sotiris Vardoulakis, Clare Heaviside, Quentin Tenailleau, Clara Squiban, António Alvarenga, Liliana Freitas, Paulo Correia, Céline Ledoux, Eva Pilot, Thomas Krafft, Hynek Pikhart, Joana Morrison, Conrad Franke, Bo Burström, Evangelia Samoli, Klea Katsouyanni, Sophia Rodopoulou, Dagmar Dzúrová, Michala Lustigová, Anna Cavallo, Nathalie Coué, Lucia Bosáková, Michal Tkáč, Hadewijch Vandenheede, Patrick Deboosere, Giuseppe Costa, Nicolás Zengarini

**Affiliations:** 1 Department of Geography and Tourism, Faculty of Arts and Humanities, University of Coimbra, Colégio S. Jerónimo, Largo D. Dinis, 3001-401 Coimbra, Portugal; 2 CEGOT-UC, Centre of Studies in Geography and Territorial Planning, University of Coimbra, Coimbra, Portugal; 3 CEG-IST, Centre for Management Studies of Instituto Superior Técnico, Universidade de Lisboa, Avenida Rovisco Pais, 1049-001 Lisbon, Portugal; 4 CEISUC, Center for Health Studies and Research, Faculty of Economics, University of Coimbra, Coimbra, Portugal; 5 ASPB, Agència de Salut Pública de Barcelona, Barcelona, Spain; 6 CIBER Epidemiología y Salud Pública (CIBERESP), Madrid, Spain; 7 Institut d’Investigació Biomèdica (IIB Sant Pau), Barcelona, Spain; 8 PHE-CRCE, Centre for Radiation, Chemical and Environmental Hazards, Public Health England, Didcot, OX11 0RQ, United Kingdom; 9 LAboratoire DYnamiques Sociales et Recomposition des espaceS (LADYSS), Paris Nanterre University, Paris, France; 10 Beuth University of Applied Sciences Berlin (BHT), Berlin, Germany; 11 Karolinska Institute (KI), Stockholm, Sweden

**Keywords:** Health equity, European Union regions, Geographic inequalities, Population Health Index, Participatory approach, Foresight, Scenarios, Policy evaluation, WebGIS

## Abstract

**Background:**

Population health measurements are recognised as appropriate tools to support public health monitoring. Yet, there is still a lack of tools that offer a basis for policy appraisal and for foreseeing impacts on health equity. In the context of persistent regional inequalities, it is critical to ascertain which regions are performing best, which factors might shape future health outcomes and where there is room for improvement.

**Methods:**

Under the EURO-HEALTHY project, tools combining the technical elements of multi-criteria value models and the social elements of participatory processes were developed to measure health in multiple dimensions and to inform policies. The flagship tool is the Population Health Index (PHI), a multidimensional measure that evaluates health from the lens of equity in health determinants and health outcomes, further divided into sub-indices. Foresight tools for policy analysis were also developed, namely: (1) scenarios of future patterns of population health in Europe in 2030, combining group elicitation with the Extreme-World method and (2) a multi-criteria evaluation framework informing policy appraisal (case study of Lisbon). Finally, a WebGIS was built to map and communicate the results to wider audiences.

**Results:**

The Population Health Index was applied to all European Union (EU) regions, indicating which regions are lagging behind and where investments are most needed to close the health gap. Three scenarios for 2030 were produced – (1) the ‘Failing Europe’ scenario (worst case/increasing inequalities), (2) the ‘Sustainable Prosperity’ scenario (best case/decreasing inequalities) and (3) the ‘Being Stuck’ scenario (the EU and Member States maintain the status quo). Finally, the policy appraisal exercise conducted in Lisbon illustrates which policies have higher potential to improve health and how their feasibility can change according to different scenarios.

**Conclusions:**

The article makes a theoretical and practical contribution to the field of population health. Theoretically, it contributes to the conceptualisation of health in a broader sense by advancing a model able to integrate multiple aspects of health, including health outcomes and multisectoral determinants. Empirically, the model and tools are closely tied to what is measurable when using the EU context but offering opportunities to be upscaled to other settings.

## Background

The increasing complexity in measuring health inequalities requires new instruments and more meaningful information at the regional and community level.

Considerable advances in knowledge have identified the key driving forces that are likely to influence health and well-being [1–6]. However, in terms of assessment methods [7, 8], there is still a lack of comparable measures able to provide a holistic assessment of population health based on the multiple determinants involved, ones which would then be applied across Europe [9], particularly at the regional and local level. It is a fact that there are significant variations amongst the factors that influence the health of European citizens [10–12]. These health variations are socially and spatially patterned, and in many cases their drivers can be traced to broader determinants such as socio-economic [4] and working conditions [5, 6], which people in Europe experience in very unequal ways [2, 13]. To characterise the health of the population currently living in European Union (EU) regions, it is crucial to consider multiple health dimensions that go beyond the health outcomes and include a range of determinants outside the healthcare sector [14, 15]. This can provide an evidence-based perspective of policies, with the potential to reduce inequalities and promote health equity [11]. Therefore, efforts towards developing models that combine multiple determinants of health, engagement of diverse stakeholders, and evidence-based policies and interventions have been stimulated and supported in the health research area [16], inclusively by the EU through its framework for funding research and innovation. These measures should be based on sound methods to enhance the potential of monitoring health and of foreseeing the impact of policies on health equity [9, 17, 18]. In our view, ‘health equity’ is understood here as “*the principle underlying a commitment to reduce and, ultimately, eliminate disparities in health and in its determinants, including social determinants*” [19].

With respect to population health determinants, it is known that health inequalities are shaped by one’s socioeconomic status, education and income level, living conditions or the physical and built environment conditions of one’s place of residence. Given that, these factors are preventable through policies and the allocation of resources and variations amongst them are deemed as inequitable and thus recognised as injustices [20]. In particular, WHO [3, 21] has acknowledged the impact of social determinants on health equity and well-being, not only by tackling personal behaviours but also by addressing life course stages, the broader society and the wider macro-level context, along with governance, delivery and monitoring systems.

Simultaneous to the discussion on the scope of health inequities, the understanding of health has evolved significantly [22], making the concept of health complex and holistic [23] and requiring that ‘what’ and ‘why’ be measured [24]. Kindig et al. [16] have suggested that, to understand the health of a society, the fundamental question to be asked is “*how we are doing – and how we can do better?*” Population health measurements have been recognised as one of the appropriate tools to support public health policy-making, monitoring and assessment by ensuring validity and cross-population comparability [9]. Summary measures built on relevant indicators are well-known instruments to provide a comprehensive picture of health and well-being [25, 26], with their multi-domain basis reflecting the complexity of people’s health. The interconnectedness of Health in All Policies [27], recognised in the Alma Ata Declaration on Primary Health Care [28] and the Ottawa Charter for Health Promotion [29], has created a new spectrum for looking at health and implied a need for an integrated approach to population health [25, 30]. Such an approach has the potential to guide policy-making and inter-sectorial action to improve health and reduce inequalities [31].

The aim of this paper is to present the ultimate outputs of the EURO-HEALTHY research project – to introduce the tools, demonstrate their capacity to measure and monitor population health across European regions, and finally present their potential to inform policy-makers and to stimulate and facilitate health equity discussions.

This paper will be of interest to policy-makers, researchers and those who wish to enhance their understanding on novel population health tools that are able to advance and promote evidence-based policy whose ultimate aim is a healthier and more equitable Europe.

## The EURO-HEALTHY project

The aim of the EU-funded EURO-HEALTHY project (which stands for ‘shaping EUROpean policies to promote HEALTH EquitY’) is to advance knowledge on policies that offer the highest potential to promote health equity across European regions (269 regions at Nomenclature of Territorial Units for Statistics level 2 (NUTS 2)) and urban areas. To achieve this goal, EURO-HEALTHY has built tools with the capacity to synthesise evidence for policy actions to address identified health inequalities and inequities across Europe [32].

**Fig. 1:**
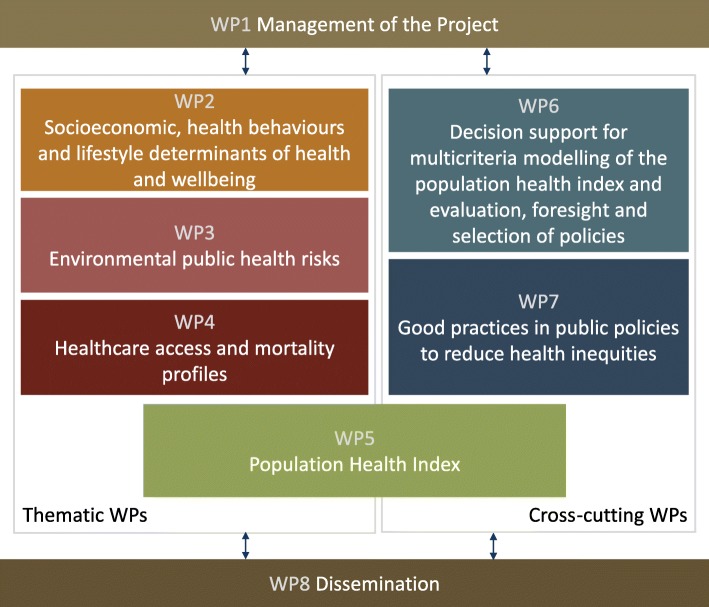
Structure of the EURO-HEALTHY project: Work Packages

From the outset, the project relied upon the collaboration of 15 multidisciplinary European institutions and involved nearly 100 stakeholders in the development and application of a sound multi-disciplinary and trans-disciplinary approach to appraise population health across EU regions. The structure of the project was based on eight Work Packages (WPs) (Fig. 1), with six WPs being directly dedicated to the scientific work and two WPs to project coordination (WP1) and dissemination (WP8). Thematic WPs (WP2, 3 and 4) conducted research to provide evidence and data on health outcomes and health determinants, including socioeconomic, health behaviours and lifestyle determinants of health and well-being factors (WP2), environmental public health risks (WP3), and healthcare access and mortality profiles (WP4). WP6 and WP7 were ‘cross-cutting’ WPs, with the former focused on designing and applying innovative, participatory processes and multi-criteria decision analysis methods to build the Population Health Index (PHI), and the latter on assessing policies and good practices in health inequalities. WP5 integrated evidence and data, engaging all the Consortium partners, including other stakeholders, in the construction and application of the PHI to 269 European regions and to 9 selected metropolitan areas.

The flagship tool is the EURO-HEALTHY PHI, a multi-dimensional and multi-level index measure which, from the lens of health equity, evaluates EU population health in two Determinants and Outcomes components. The PHI is further divided into sub-indices by area of concern, health dimension and indicators, and is designed to be applied across all regions of the 28 EU Member States [32] and to selected metropolitan areas [33].

To enlighten the discussion on policies to promote health equity in Europe, the PHI model was used as the starting point to develop the EURO-HEALTHY Scenarios for Population Health Inequalities in Europe for 2030 [32]. The rationale for developing the scenarios was two-fold – policy-makers not only need reliable tools to help them holistically evaluate the policies’ benefits in terms of their feasibility and power issues, but they must also reflect upon and assess how future events may affect health inequalities and which policies may produce adverse outcomes [34–36].

The bridge between academia, policy-makers and civil society was promoted by the EURO-HEALTHY consortium in two main forms – by using large-scale participatory approaches in the development of the EURO-HEALTHY PHI and of the EURO-HEALTHY scenarios, and by making mapping tools available for the dissemination of the results of applying the PHI to European regions amongst policy-makers and a wider audience, including civil society. Recognising that the Geographical Information Systems (GIS) facilitate both the compilation, analysis and dissemination of large and multi-dimensional information sets [37], the project has developed a WebGIS platform called *healthyregionseurope* (https://healthyregionseurope.uc.pt). This online tool allows for the space–time analysis, monitoring and comparison of population health (current and future) at 269 NUTS 2 European regions, and for 9 selected metropolitan areas (Athens, Barcelona, Berlin, Brussels, Lisbon, London, Prague, Stockholm and Turin).

## Data and methods

The project collected and systematised a vast amount of data for 80 indicators chosen for their relevance in assessing population health in Europe at three geographical levels – national (countries), regional (NUTS 2) and metropolitan (for selected municipalities) – and for the years 2000–2015. These data were aggregated in an online platform accessible at https://eurohealthydata.uc.pt that stores a wide range of data collected during the project and available for consultation and download. It can support researchers in their data analyses by facilitating access to a database with multiple indicators to investigate further inequalities and inequities in health across Europe.

### The construction of EURO-HEALTHY PHI

The PHI structure is based on a multi-criteria model that was built with the MACBETH (Measuring Attractiveness by a Categorical Based Evaluation Technique) socio-technical approach [38] and makes use of Multi-Criteria Decision Analysis and value measurement concepts, integrating the technical elements of a multi-criteria value model and the social elements of interdisciplinary and participatory processes [39].

The set of indicators used within the PHI was informed via a participatory process in which experts and other stakeholders accessed updated scientific evidence and judged the relevance of indicators identified by the Consortium members within the WPs to appraise population health at the EU regional level [15]. A multidisciplinary group of 81 experts was involved in the participatory processes, from the structuring of the PHI multicriteria model (areas of concern, dimensions and indicators) to the evaluating phases (weights, value functions). These 51 Consortium members and 30 other stakeholders reflected not only a wide range of expertise but also Europe’s diversity [15], as the group comprised representatives of national, regional and local authorities, advisors and technicians, international bodies, political parties, healthcare professionals, and urban planners (Table 1). The participatory processes are detailed below in the section describing stakeholders’ involvement.

**Table 1:** Composition and characteristics of the panel of experts involved in the construction of the Population Health Index

Characteristic	No.
Total	81
Gender	
Male	37
Female	44
Type of panellist	
Expert	51
Stakeholder	30
Field of expertise	
Economics and health systems	9
Environmental health, ecological systems, sustainability	15
Epidemiology, social medicine and public health	29
Health geography, demography and sociology	28
Region of Europe	
Northern Europe	16
Western Europe	18
Eastern Europe	12
Southern Europe	35

**Fig. 2:**
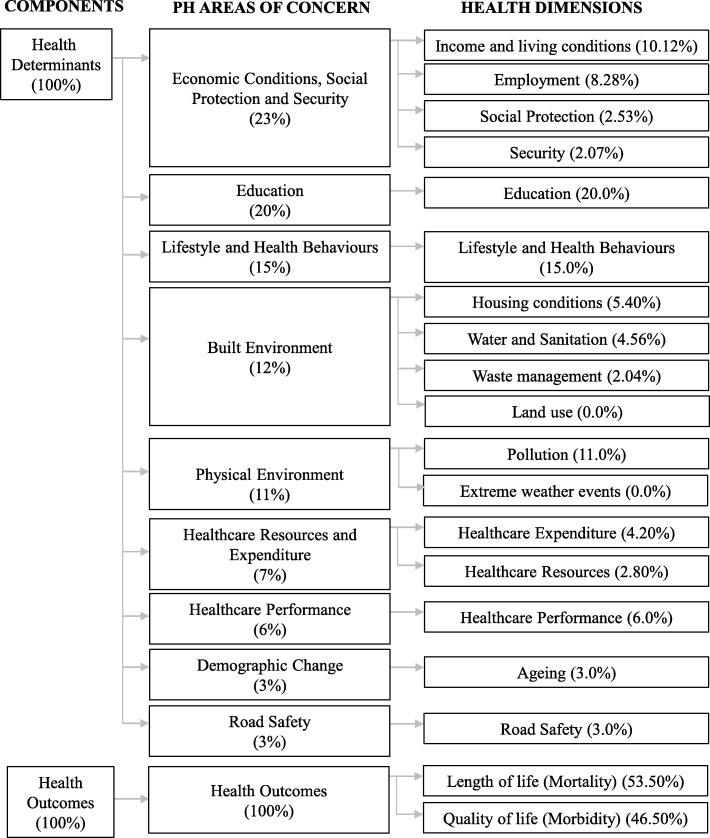
Population Health Index structure (entailing Components, population health areas of concern, health dimensions and respective weights)

Presenting a bottom-up hierarchical structure, the PHI provides an evidence-based approach to analyse inequalities within a chained sub-index structure (Fig. 2) headed by Health Determinants and Health Outcomes indices. These indices are divided into ten sub-indices, corresponding to the main areas of concern for population health, namely (1) economic conditions, social protection and security, (2) education, (3) demographic change, (4) lifestyle and health behaviours, (5) physical environment, (6) built environment, (7) road safety, (8) healthcare resources and expenditure, (9) healthcare performance, and (10) health outcomes. These sub-indices are further divided into health dimensions and indicators. Each area of concern integrates a set of population health dimensions which are independent evaluation axes for appraising population health and are made operational by one or more indicators (Table 2).

**Table 2:** List of indicators included in the EURO-HEALTHY Population Health Index model, grouped by population health area of concern and health dimension

Population health area of concern	Health dimension	Indicators
Economic conditions, social protection and security	Employment	Unemployment rate (%)
		Long-term unemployment rate – 12 months and more (%)
	Income and living conditions	Disposable income of private households per capita (Euro per inhabitant)
		People at risk of poverty or social exclusion (%)
		Disposable income ratio – S80/S20 (ratio)
	Social protection	Expenditure on care for the elderly (% of GDP)
	Security	Crimes recorded by the police (per 100,000 inhabitants)
Education	Education	Population aged 25–64 with upper secondary or tertiary education attainment (%)
		Early leavers from education and training (%)
Demographic change	Ageing	At risk of poverty rate of older people – aged 65 years and over (%)
		Ageing index (ratio)
Lifestyle and health behaviours	Lifestyle and health behaviours	Adults who are obese (%)
		Daily smokers – aged 15 and over (%)
		Pure alcohol consumption – aged 15 and over (litres per capita)
		Live births by mothers under the age of 20 (%)
Physical environment	Pollution	Annual mean of the daily PM_2.5_ concentrations (μg/m^3^)
		Annual mean of the daily PM_10_ concentrations (μg/m^3^)
		Greenhouse gases (total tonnes of CO_2_ eq. emissions per capita)
		Population exposed to traffic noise – (Lden 55–59 db, during day) (%)^a^
	Extreme weather events^a^	Population affected by flooding (per 1,000,000 inhabitants)^a^
Built environment	Housing conditions	Average number of rooms per person
		Households without indoor flushing toilet (%)
		Households without central heating (%)
	Water and sanitation	Population connected to public water supply (%)
		Population connected to wastewater treatment plants (%)
	Waste management	Recycling rate of municipal waste (%)
	Land use^a^	Population density (inhabitants/km^2^)^a^
Road safety	Road safety	Victims in road accidents – injured and killed (per 100,000 inhabitants)
		Fatality rate due to road traffic accidents (per 1000 victims)
Healthcare resources and expenditure	Healthcare resources	Medical doctors (per 100,000 inhabitants)
		Health personnel – nurses and midwives, dentists, pharmacists and physiotherapists (per 100,000 inhabitants)
	Healthcare expenditure	Total health expenditure (PPS$ per capita)
		Private households’ out-of-pocket expenses on health (% of total health expenditure)
		Public expenditure on health (PPS$ per capita)
Healthcare performance	Healthcare performance	Hospital discharges due to diabetes, hypertension and asthma (per 100,000 inhabitants)
		Amenable deaths due to healthcare (standardised death rate per 100,000 inhabitants)
Health outcomes	Length of life (mortality)	Life expectancy at birth (years)
		Infant mortality (per 1000 live births)
		Preventable deaths (standardised death rate per 100,000 inhabitants)
	Quality of life (morbidity)	Self-perceived health less than good (%)
		Age-standardized disability-adjusted life year rate (per 100,000 inhabitants)
		Low birth weight (%)

*Lden* day–evening–night noise level, *PPS* purchasing power standards

^a^ Dimensions and indicators included in the Population Health Index model (conceptual) but not used in its application (adjusted) to the 269 NUTS 2 regions, due to lack of data

More details on the methodology applied to build the PHI are given in Bana e Costa et al. [39] and Santana et al. [40].

### EURO-HEALTHY scenarios

Technically, the approach to building EURO-HEALTHY scenarios required a process that would create such scenario structures to include a large number of drivers and combine a group elicitation method with the Extreme-World method [41, 42]. The social side of the approach required a combination of face-to-face and non-face-to-face participatory processes to obtain the views and opinions of health stakeholders and experts. A Web-Delphi process was conducted with a large and diverse panel of experts and other stakeholders and was established to generate ideas for potential causes of future changes as well as to inform which drivers were relevant for future population health inequalities (departing from current health inequalities across European regions). Drivers identified by the panellists were complemented with future-oriented evidence and organised under the PESTLE categories (Political, Economic, Social, Technological, Legal and Environmental). This set of data was the starting point from which a strategic small group worked to organise the drivers into three scenario structures in a (face-to-face) workshop process format. The process was finalised by developing scenario narratives for the three plausible scenario structures [42].

The detailed description of this process is available in Alvarenga et al. [43].

### Evaluating and selecting urban policies under EURO-HEALTHY scenarios

The multi-methodology uses Multi-Criteria Decision Analysis to appraise policies on a common basis and, within the case study, was used to engage 33 local stakeholders in a series of face-to-face workshops to identify critical situations for equity in the Municipality of Lisbon. In this process, a strategic group of 16 stakeholders (policy-makers, health professionals, urban planners, social security practitioners, representatives of civil society) was engaged in a 2-day decision conferencing process to evaluate the potential of a set of policies to improve overall health and reduce health inequities. The impact of policies was valued according to their contribution to priority intervention axes corresponding to eight of the above-mentioned PHI areas of concern and to the comparative added value of improving each of these intervention axes. The feasibility of policies was assessed in light of the EURO-HEALTHY scenarios.

### WebGIS – healthyregionseurope

EURO-HEALTHY developed an open access user-friendly WebGIS platform (http://healthyregionseurope.uc.pt) to provide both data and a snapshot of European population health over multiple dimensions and geographical scales – at the regional level, for 269 NUTS 2 regions of the 28 EU countries, and at the municipal level, for 540 municipalities of 9 selected metropolitan areas (Athens, Barcelona, Berlin, Brussels, Lisbon, London, Prague, Stockholm and Turin).

**Fig. 3:**
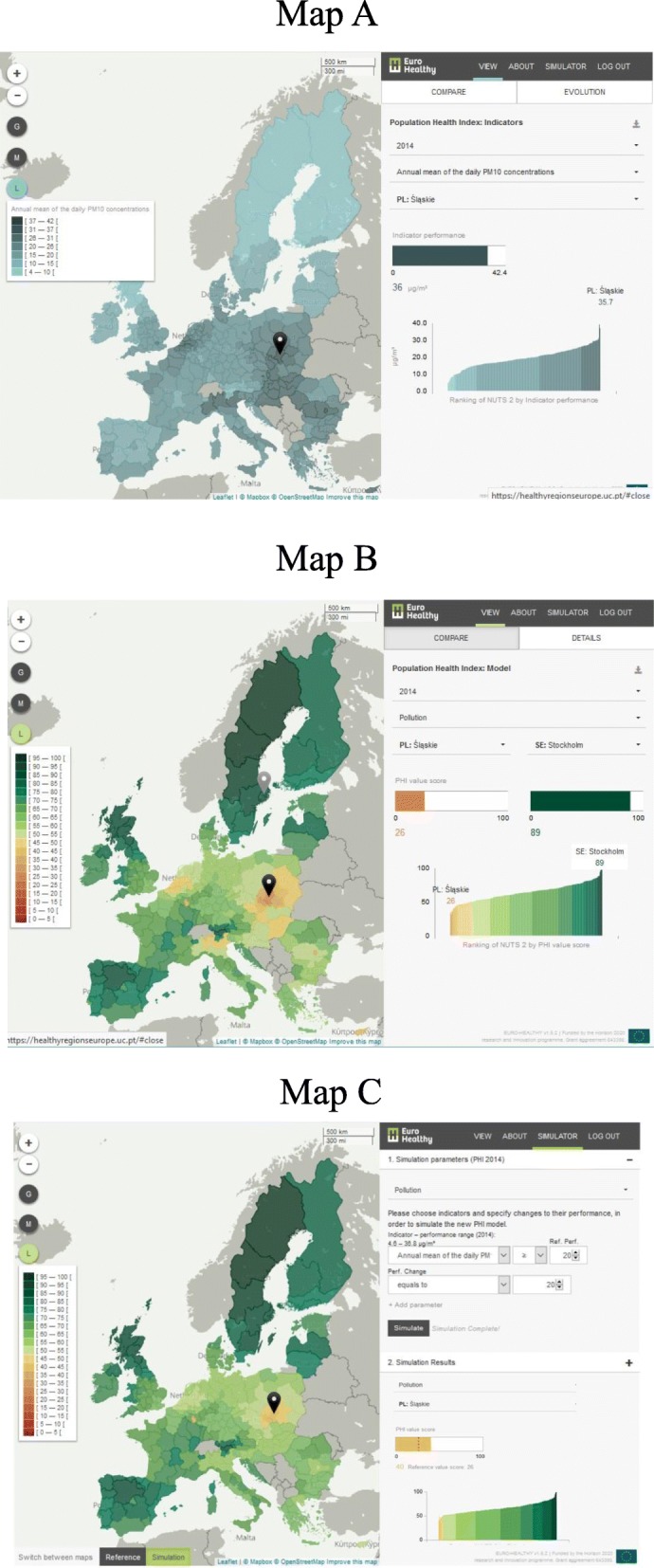
Screenshots from the WebGIS *healthyregionseurope*. Note: Map **a** shows the tab view for the Population Health Index (PHI) indicators, in terms of performance (illustrative example is presented for the indicator “Annual mean of the daily PM_10_ concentrations” where the performance of one region – Śląskie, Poland – is shown). Map **b** shows the tab view for the PHI model, in terms of value-score (illustrative example is presented for the health dimension “Pollution” where the value-scores of two regions – Śląskie, Poland, and Stockholm, Sweden – are being compared). Map **c** shows the tab for the Simulator (illustrative example is presented for the simulation conducted in the health dimension “Pollution”, where a change of performance on the indicator “Annual mean of the daily PM_10_ concentrations” generated new value scores; a specific example of value improvement can be seen for the region Śląskie – Poland)

The platform was built to serve not only as a visualisation tool, but also to enable the exploration and analysis of geographical patterns of the PHI value-scores (ranging for each indicator from 0 to 100, with 0 representing the lowest level of population health and 100 the highest level of population health) for the year 2014 (Fig. 3).

## Results

The EURO-HEALTHY PHI can be read as a ‘composite measure’, ‘composite index’ or ‘composite indicator’ based on a clear conceptual framework, integrating the different elements of population health and capable of considering multiple aspects delivered as an aggregated score. The EURO-HEALTHY project sustained the added-value of developing such a holistic measure, integrating indicators from multiple health dimensions across EU countries, regions and selected metropolitan areas [32].

The PHI follows a population health approach as described and popularised by Kindig and Stoddart [44], taking the view that population health should be measured considering the “*health outcomes and their distribution within a population, the patterns of determinants that influence such outcomes, and the policies that influence the optimal balance of determinants*”. The underlying assumption focuses on improving the health of the population rather than that of individuals, and on reducing health inequalities through actions that target the determinants of health.

In line with this, the EURO-HEALTHY PHI was designed as a comprehensive tool to enable evidence-based policy-making by (1) allowing for the measuring and monitoring of the overall health and well-being of regional European populations; (2) accounting for the multi-dimensional nature of health determinants; (3) foreseeing and discussing the impact of multi-level policies that can influence population health and geographical health inequalities in Europe; and (4) providing a basis for multilevel policy dialogue on health and health equity.

The EURO-HEALTHY PHI was applied to analyse population health and identify geographical health inequalities in 269 NUTS 2 level regions, 9 selected metropolitan areas (Athens, Barcelona, Berlin, Brussels, Lisbon, London, Prague, Stockholm and Turin) and in 2 case studies (Lisbon and Turin). The PHI outputs offer different ways of looking at health inequalities in Europe. For instance, they provide opportunities to analyse how European Structural and Investment Funds can be used to reduce population health inequalities and enable policy dialogue at national and regional levels [45]. In addition, when the PHI is regularly updated, it becomes a valuable resource for policy monitoring and evaluation.

In the interest of developing foresight tools for policy improvement, a key goal of the project was to build EURO-HEALTHY scenarios to inform potential drivers with a means to affect future health and health inequalities across European regions and to understand the extent to which future evolutions can be triggered by today’s actions [46]. Following these objectives, EURO-HEALTHY produced health inequalities scenarios for 2030 through a novel socio-technical approach that combined present and future-oriented evidence with the perspectives and values from a diverse group of experts and other stakeholders across Europe within a structured process.

Accordingly, three EURO-HEALTHY scenarios for 2030 were produced [42] – (1) the ‘Failing Europe’ scenario as a worst-case (although plausible) scenario, including all driver configurations leading to enlarging the population health inequalities; (2) the ‘Sustainable Prosperity’ scenario based on the best-case scenario structure, including all configurations leading to decreasing the population health inequalities; and (3) the ‘Being Stuck’ scenario, which reflects a situation where the EU and EU Member States maintain the status quo and are ‘stuck’ with those specific difficulties that keep Europe from moving forward such as generating new consensus or common policies.

Two scenarios – ‘Sustainable Prosperity’ and ‘Failing Europe’ – were tested in a real exercise conducted within the scope of the Lisbon case study to formulate and assess policies to improve urban health inequalities, which we further describe.

Aligned with the methods used to build the PHI and scenarios, EURO-HEALTHY developed a transparent multi-methodology to inform policy appraisal and selection. Underlying the multi-methodology is (1) the recognition of the importance of policy-makers and other stakeholders’ engagement in the policy appraisal process; (2) the need for policy selection accounting for policy feasibility and other implementation issues; (3) the acknowledgment that foreseeing the impact of policies involves uncertainty and that scenarios are a tool to deal with uncertainty in the policy appraisal process; (4) the recognition that health public policies aim to achieve multiple objectives, including maximising health gains and reducing inequalities; and (5) the acknowledgment that indices are powerful tools for assessing the impact of policies.

The application of this multi-methodology to evaluate local policies in the Lisbon case study was successful in illustrating, firstly, which policies have higher potential to improve overall health and, secondly, how the local policies’ feasibility can change according to different European scenarios. Specifically, European scenarios have been shown to have an important impact on the capacity of the Lisbon municipality to implement certain policies and to raise awareness of those individuals involved in the decision-making process as to the policies’ risks and opportunities [47]. Furthermore, the participatory component of the multi-methodology demonstrated how interaction between local stakeholders can be facilitated given the emphasis on the different perspectives on health equity and its indicators within structured and transparent formats.

The WebGIS offers a set of dynamic features for users exploring population health in Europe (1) analysing and comparing the regional performances and value-scores in an aggregated and disaggregated way, following a value tree index structure (with sub-indices for components, areas of concern, dimensions and indicators) (Fig. 3, maps a and b), and (2) simulating changes in the performances of indicators (as if considering the impact of policies) to understand the potential effect of policies on the regional population health scores and thus on regional inequalities across Europe. For instance, the simulator web functionality provides evidence on the actual inequalities across EU regions and allows the user to change one or a set of indicators’ performances, considering different policy strategies (e.g. reducing the levels of PM_10_ of all regions whose current performances are above 20 μg/m^3^ to the reference value of 20 μg/m^3^) and generating new population health scores (Fig. 3, map c). In addition, maps show the regions and countries that benefit from a given policy or from a package of policies.

These WebGIS platform functionalities support the transfer of knowledge from scientists to policy-makers and to the public in general, as it communicates fundamental information in a user-friendly way to help those concerned with understanding and reducing health gaps in Europe.

The WebGIS is a web database platform that was specifically designed to support researchers in data analysis and to investigate inequalities and inequities in health across EU regions. The complementary *eurohealthydata* platform is accessible at https://eurohealthydata.uc.pt and is password protected (passwords assigned upon request via the website) and aggregates 80 indicators collected at 3 geographical levels – national (countries), regional (NUTS 2) and metropolitan (municipalities) – for the period 2010–2015. This web-based data platform has the capacity to store and download all data (metadata, alphanumerical and geographical data) collected in the project. Both platforms were built using open source software.

## Discussion

From its inception, the project was conceived to represent widely shared principles of the EU with respect to economic, social and territorial cohesion as a way to promote the well-being of all EU citizens. The production of reliable evidence to inform the European efforts to obtain these values provide the basis of the research activities. We herein emphasise the key features and the potential for learning from EURO-HEALTHY research.

### Evidence and tools supporting policy-makers and stakeholders to address health inequalities on multiple scales

The information generated by the PHI not only allows for a deeper understanding of which factors influence the overall health of a specific EU region or groups of regions, but it can also provide guidance for the evaluation and selection of policies with the greatest potential to address inequalities between regions.

The regional population health scores can be analysed in various forms, e.g. by grouping regions with a similar profile in terms of performance in a specific dimension or indicator (clusters), by measuring statistically significant differences between groups of regions (e.g. by categories of regions eligible to receive Cohesion Policy funds) and by identifying which dimensions constitute areas for improvement across all EU regions. By utilising the Health Determinants Index and its dimensional sub-indices, policy-makers at different levels of decision-making (European, national and regional) are able to examine the relationships between health determinants, health outcomes and policies from different sectors. The PHI works as a basis for understanding which policies (from different sectors and competing for resources) have the highest potential to improve health (e.g. in a specific region or group of regions of the same country) and to decrease health inequalities (e.g. between regions of the same country or between EU regions). Further analysis can also be done on what policies have contributed to the current population health scores in selected regions.

From the perspective of policy evaluation, the PHI multicriteria model can be used both as an instrument to design and select policies on an ex ante basis as well as ex post, monitoring the impact of policies across time and to perform cost-effectiveness analyses. This would require a dynamic update of the PHI, which implies feeding the model with up-to-date data and, potentially, the inclusion of new indicators reflecting novel research and evidence pointing out other factors linked to local priorities or global trends. It should be noted that the updating of the PHI model offers opportunities but also presents certain constraints to be overcome, namely those related to data availability in some dimensions. Data gaps at the regional level on several indicators led to some adjustments between the conceptual model and the model implemented and available via the WebGIS. Issues with availability and quality of the indicator data at sub-national levels should be fundamentally addressed in order to obtain the full potential of this tool [48].

One of the strengths of using the PHI and the methods employed in its construction is the involvement of stakeholders and experts whose knowledge is beneficial to the robustness and applicability of the results. Innovative policy design, using the PHI in conjunction with the EURO-HEALTHY scenarios, can be introduced to tackle health inequalities across multiple domains and levels. Together, these tools inform on current population health variations in the EU, on the multiple factors underlying those variations (drivers) and on how overall health will evolve if those factors are changed positively or negatively.

The PHI results can identify what the problems are and where action is most needed. Policy-makers and stakeholders can easily access this information through a WebGIS platform that communicates the PHI in a very interactive way, pointing to health inequalities between regions and across multiple dimensions.

EURO-HEALTHY considered that maps displaying health information in a simple, clear way are important analytic tools for decision-makers in their efforts to support planning, develop equitable policy and assess equity [49]. GIS have become tools of considerable usefulness [50] in monitoring diseases [51], detecting the differences and similarities in population health [52], and in enabling overall understanding of health distribution in the population [53] and of the relationship between health and space [54, 55]. The adoption of a place-based approach and a multi-methodology (including scenarios) to evaluate local policies in the Lisbon case study unlocked the potential for transformative change in the way of formulating and evaluating policies across sectors that go beyond the health sector. This experience could be transferred to other cities with the necessary adjustments in terms of panel formation, indicators and the policies at stake.

### Stakeholders’ involvement through large-scale participatory processes

At the heart of the project was the force of continued stakeholder engagement alongside innovative methods to develop the EURO-HEALTHY tools. Over a 3-year journey, the project has, in a transdisciplinary way, integrated more than 150 stakeholders and experts from 15 European countries, with these being involved directly in research design from the beginning of the project. This process of sharing ideas, bringing European countries together, and learning from each other strengthened the solidarity of the project’s action. Stakeholders were identified by the EURO-HEALTHY consortium partners and selected based on a variety of characteristics, namely (1) their ability to influence policy at various decision-making levels (national, regional and metropolitan), (2) their scope for intervention (public sector, private sector and civil society), (3) their area of work (e.g. environment, public health, urban planning, groups at risk), and (4) geographic location (meant to reflect Europe’s diversity) [56].

Stakeholders and researchers were regularly involved in the project research activities to develop the EURO-HEALTHY tools, produce knowledge and evidence, and mostly build a bridge of communication between scientists and policy/decision-makers [56]. Web-based Delphi panels, decision conferences and face-to-face workshops were successful formats for interacting with stakeholders and researchers to collect their insights and views on (1) the relevant indicators to evaluate and monitor regional European population health (Web-Delphi for selecting indicators for the PHI) [15]; (2) the importance of closing gaps in the performance of distinct indicators across EU regions (Web-Delphi for weights) [39]; (3) added value to population health from improving performance along the indicator range (Web-Delphi for value functions) [39]; (4) drivers for future population health inequalities scenarios (Web-Delphi for scenario building) and for population health inequalities scenarios in Europe (workshops) [42]; and (5) formulation and evaluation of policies with the highest potential to improve population health and to reduce inequities at the urban level, namely in the Lisbon and Turin case studies (workshop and decision conferences) [35].

EURO-HEALTHY web-based Delphi participatory processes have proved to be a convenient and time-efficient method for multi-level stakeholders. This has also been confirmed in other health-related studies [14] and has served to generate useful information to help smaller groups in the construction of the EURO-HEALTHY PHI model and EURO-HEALTHY scenarios, and in the evaluation of policies.

The involvement of a transdisciplinary panel of researchers and stakeholders in the process of shaping the EURO-HEALTHY tools added diverse points of view, which consequently validated the holistic perspective of looking at health. This approach was crucial in securing sustainable engagement from stakeholders in the project activities, enhancing their understanding of the drivers of health inequalities in Europe, and strengthening their will, interest and power to promote change via their future decisions [15]. It served as a catalyst for an extended dialogue as to which policies produce the highest benefit in promoting more equitable and healthy environments at different levels (national, regional and local), while bearing in mind the impact that different policies can have on population health and health equity [57]. In this sense, the project represented “*the science of stakeholder engagement in research*” – a term coined by Goodman and Thompson ([58], p. 489), where they strongly advocate for enhancing efforts towards meaningful stakeholder engagement that will contribute to both research synergy and achieving results that would otherwise be impossible via isolated actions alone. The Lisbon and Turin case studies have shown that stakeholder engagement is fundamental to promoting consensus and establishing priorities for intervention at the local scale to reduce unjust health inequalities in transparent formats [32].

### ‘Learning networks’ to reduce health inequities across Europe

It is a stated challenge for the EU to take action centrally to address the highly localised, spatially patterned health issues that are often heavily shaped by local and regional circumstances. Featuring broad-ranging data on a variety of countries, regions and metropolitan areas, and gathered and utilised in one measure, the PHI facilitates the joint learning process, encourages conversation and dialogue, and supports the identification of relevant partners (regions) tackling similar problems. In this context, the evidence provided by the project, and specifically by the PHI, has the potential to create windows of opportunity, ones which could evolve into the establishment of ‘learning networks’ – mechanisms supporting informed and localised actions on multiple health determinants to mitigate the unjust inequalities in a multi-dimensional environment. This process would assist in advocating, stimulating and facilitating those actions that can promote equity in European health through promoting knowledge transfer between scientists and decision-makers. The creation of these ‘learning networks’, whether between regions and/or cities, would highly benefit from EU funding, namely via European Structural Investments Funds, which provide the adequate framework to boost synergies and implement such regional networking. This would create greater opportunities for regional convergence and for documented good practices with the potential to improve other low-performing localities. This value takes on significant meaning in decision-making and decision support to improve equity and facilitate successful action.

## Further research

Further research on developing technological platforms for expert and stakeholder engagement should be emphasised given the relevance of considering different points of view as well as disseminating scientific evidence and reliable data. In this context, the Web-Delphi participatory processes applied in the EURO-HEALTHY project have proved to be an inclusive and effective way to collect information from an expanded number of geographically dispersed experts and stakeholders. Yet, a certain amount of sensitivity bias related to the composition of the panels was expected due to the different backgrounds and geographical contexts in which experts and stakeholders live and work. In theory, the experts’ judgments may not be in line with the context where the decision-making process is to be integrated. Therefore, in order to reduce the expert’s bias in future studies, this issue should be addressed.

The development and application of the PHI model at other geographic levels (e.g. local) and settings (e.g. North America, Africa) requires further research on adjusting the model to the specific contexts. Conducting participatory processes with local panels of stakeholders and other experts, for example, by defining adequate indicators that reflect the realities of local conditions and the respective weights and value functions, are considered imperative.

The EURO-HEALTHY PHI may also serve as a starting point and useful tool to initiate a dialogue with local stakeholders and policy-makers about which priority intervention fields each locality should address in the interest of improving overall population health. This would be possible by further extending the evaluation of policies conducted in the Lisbon and Turin case studies to other case studies and by creating tools and knowledge to integrate policy evaluation with uncertainty and scenarios.

The EURO-HEALTHY tools were built under the points of view and perspectives of a panel of researchers and stakeholders taking into account the available data at the present time. In the future, it is absolutely necessary to continuously monitor data on health determinants and health outcomes in order to update the models of PHI and scenarios. The set of relevant indicators and respective weights could then change, reflecting alterations driven by policies or new societal challenges. Explicit attention should be given to social, economic and environmental determinants that shape health equity. These indicators are fundamental to inform policy and monitor its effectiveness. Currently, despite significant advances in data harmonisation at the EU level (e.g. Eurostat), there is still room for improved data to monitor and assess specific health determinants [48] that shape health outcomes at different geographical levels in a comparable way [59]. The benefit of using the PHI in the future will be accentuated if there is greater alignment of the data collected from different data sources (in the current PHI not all data were available at the same time and spatial resolution). Efforts should be intensified to collect and harmonise data at the sub-national level with the goal of narrowing the gaps in data amongst and within the EU Member States [48].

## Conclusions

The EURO-HEALTHY project achieved its goal in providing a comprehensive evaluation of health inequalities across Europe and ultimately incorporating innovative ways of supporting the analysis and selection of policies with the potential to shape healthier settings. The project has delivered tools – mostly based on the PHI – which are scientifically and methodologically robust and innovative and yet simple and user-friendly. The open-access WebGIS platform offers an easy and interactive way to visualise and analyse the population health variations across EU regions and metropolitan areas in a way that can enable more informed health advocacy across Europe. The highly participatory component proved to be paramount for integrating multiple views and facilitating information exchange amongst the involved stakeholders and experts.

At the same time, the data and evidence that was generated to feed these tools provide opportunities for comparability across different geographical scales, which is an appeal to reinforce efforts to collect, harmonise and use data, namely at sub-national level.

The EURO-HEALTHY tools should be used, particularly by policy-makers, to support decision-making in relation to those policies with the highest potential to improve health equity in Europe and to further monitor the impact of the policies of today as well as those of the future.

## Data Availability

The datasets generated and analysed during the current study are stored in the *eurohealthydata* repository (https://eurohealthydata.uc.pt) and are available from the corresponding author upon reasonable request.

## Abbreviations

EU: European Union
EURO-HEALTHY: Shaping EUROpean policies to promote HEALTH equitY
GIS: Geographic Information System
NUTS 2: Nomenclature of Territorial Units for Statistics level 2
PHI: Population Health Index
WPs: Work Packages
